# Effect of serum metabolites on the risk of iridocyclitis: a bidirectional Mendelian randomization study

**DOI:** 10.1038/s41598-024-61441-4

**Published:** 2024-05-08

**Authors:** Xuyan Zou, Yijie Lu, Yao Tan

**Affiliations:** 1 Changsha Aier Eye Hospital, Changsha, Hunan Provine 410015 China; 2 Shenzhen Aier Eye Hospital, Aier Eye Hospital, Jinan University, Shenzhen, Guangdong Provine 518000 China; 3 Department of Ophthalmology, The Third Xiangya Hospital, Central South University, No. 138 Tongzipo Road, Yuelu District, Changsha, Hunan Provine 410013 China

**Keywords:** Iridocyclitis, Serum metabolites, Mendelian randomization, LDSC, Causal association, Clinical genetics, Genetic association study, Eye diseases

## Abstract

Previous research has linked serum metabolite levels to iridocyclitis, yet their causal relationship remains unexplored. This study investigated this potential causality by analyzing pooled data from 7824 iridocyclitis patients in a Genome-Wide Association Study (GWAS) using Mendelian randomization (MR) and linkage disequilibrium score regression (LDSC). Employing rigorous quality control and comprehensive statistical methods, including sensitivity analyses, we examined the influence of 486 serum metabolites on iridocyclitis. Our MR analysis identified 23 metabolites with significant causal effects on iridocyclitis, comprising 17 known and 6 unidentified metabolites. Further refinement using Cochran's Q test and MR-PRESSO indicated 16 metabolites significantly associated with iridocyclitis risk. LDSC highlighted the heritability of certain metabolites, underscoring genetic influences on their levels. Notably, tryptophan, proline, theobromine, and 7-methylxanthine emerged as risk factors, while 3,4-dihydroxybutyrate appeared protective. These findings enhance our understanding of the metabolic interactions in iridocyclitis, offering insights for diagnosis, unraveling pathophysiological mechanisms, and informing potential avenues for prevention and personalized treatment.

## Introduction

Iridocyclitis, an acute inflammation of the iris and ciliary body, is the most common type of uveitis, present in 85% of patients. It is estimated to cause up to 10% of legal blindness in the United States^1^. Typical clinical features of iridocyclitis include eye redness, pain, blurred vision, photophobia, and miosis^2^. Typically, iridocyclitis is a benign condition, but if left unrecognized and untreated, it can also lead to severe consequences such as cataracts, secondary glaucoma, and ultimately blindness^3,4^. A large number of basic studies have now shown that uveitis is associated with genetic susceptibility, T and B cell involvement, cytokine and chemokine labeling and signaling pathways, and environmental influences^5–8^. Elucidating the etiology of iridocyclitis may be challenging because there is a great deal of variability in these mechanisms. However, this elucidation remains important, and we need a more complete understanding of the biochemical and genetic pathways involved in the onset and progression of iridocyclitis.

A novel technology called metabolomics can be used to analyze the metabolites of proteins and genes and gives substantial information on the dynamic metabolic reactions of living systems to pathogenic stimuli or genetic alterations^9^. These metabolic profiles are particularly accurate predictors of the pathophysiological properties of a tissue or the chemical makeup of an organism that cannot be determined through genetics or proteomics^10^. Metabolomics studies can identify potential differential metabolites between iridocyclitis patients and healthy volunteers, reveal metabolic dysregulation and its potential role in the pathogenesis of the disease, and search for potential biomarkers and metabolic pathways to increase understanding of iridocyclitis. However, in terms of the causal relationship between specific metabolites and iridocyclitis, further exploration and studies are still needed.

Mendelian randomization (MR) is a research technique that determines whether exposure has a causal impact on outcomes by using genetic variants found in genome-wide association studies (GWAS) as instrumental variables (IVs)^11,12^. Gregor Mendel created the law of independent classification, which results in a random distribution of these genetic variants throughout the population. Mendelian randomization is based on the random assignment of genetic variants at the time of conception^13^. These polymorphisms are typically unaffected by confounding variables or reverse causation, so variations in risk factors account for the disparities in outcomes between those who possess these variants and those who do not. This makes MR a time- and money-saving strategy for identifying potential connections between exposure and result, together with the simplicity of access to a large number of potential IVs to represent the exposure of interest^14^. In addition to Mendelian randomization, our study employs linkage disequilibrium score regression (LDSC) to further elucidate the genetic architecture underlying iridocyclitis. LDSC is a methodological approach designed to quantify the genetic correlation between traits or diseases by leveraging genome-wide summary statistics^15,16^. By assessing the extent to which single nucleotide polymorphisms (SNPs) common to two traits influence their genetic correlation, LDSC provides insights into the shared genetic etiology between diseases and traits. In this study, we utilize the integration of MR and LDSC to uncover potential genetic and metabolic pathways in iridocyclitis, reveal protective and risk factors, and deepen our understanding of the pathophysiology of iridocyclitis.

## Materials and methods

### Metabolite data source overview

**Figure 1:**
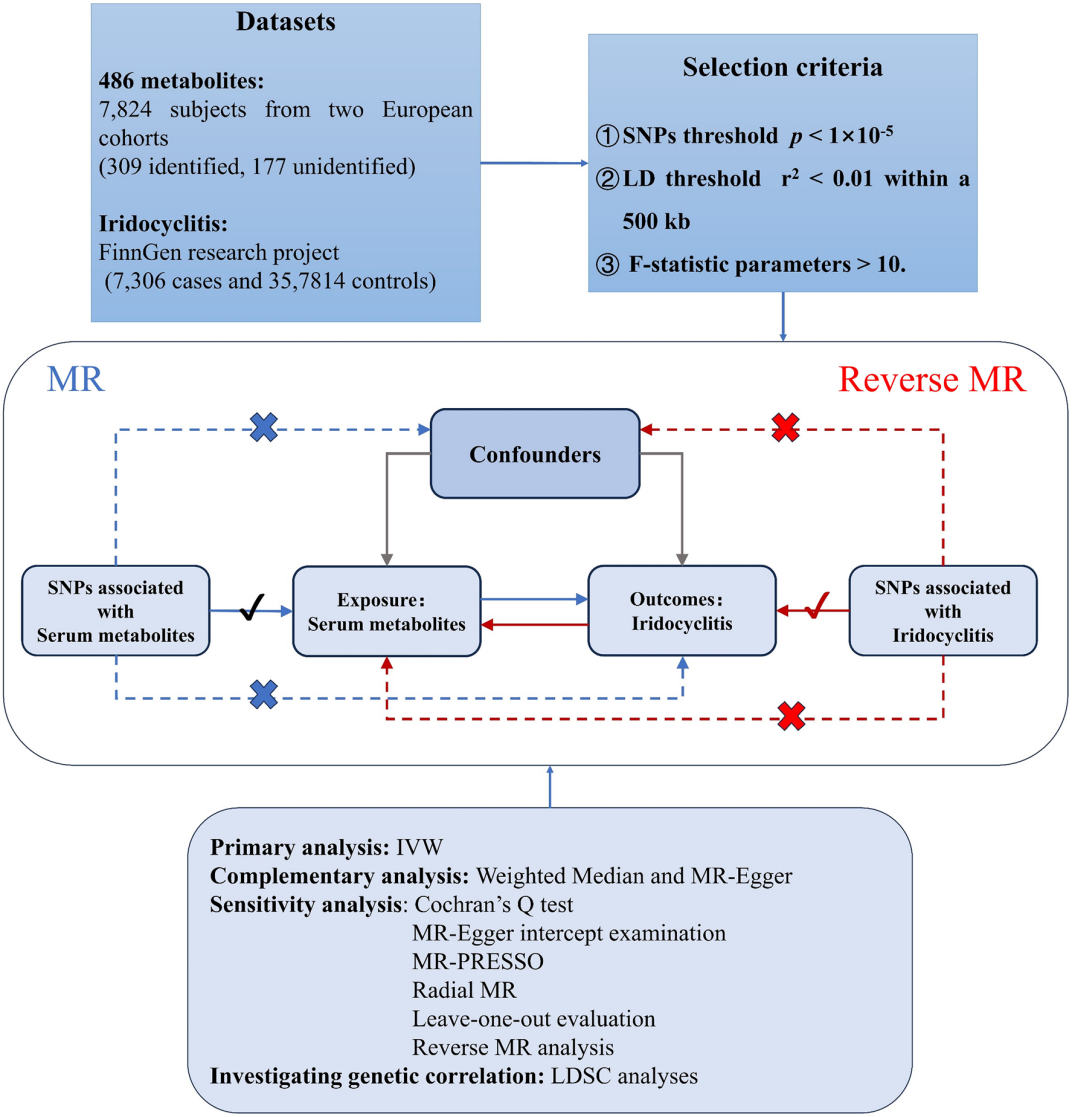
Flow chart of Mendelian randomization study. The entire workflow of Mendelian randomization analysis. *MR* Mendelian randomization, *IVW* inverse variance weighted, *LDSC* linkage disequilibrium score regression.

Our MR research, portrayed in Fig. 1 and conforming to the STROBE-MR checklist^17^, utilized genetic information concerning serum metabolites from the metabolomics GWAS server. The comprehensive non-targeted metabolomics GWAS conducted by Shin et al. informed the study significantly, identifying genetic determinants for 486 serum metabolites^18^. This probing involved 7824 subjects from two European cohorts: the KORA F4 research in Germany and the UK Twin Study. All subjects granted informed consent, with ethical approval from their respective local committees. Fasting serum underwent non-targeted mass spectrometry examination, with metabolic assessments standardized by Metabolon, Inc. Following rigorous quality control, 486 metabolites (309 identified, 177 unidentified) were scrutinized. The metabolites were classified into eight biochemical categories according to the Kyoto Encyclopedia of Genes and Genomes (KEGG) database^19^. Roughly 2.1 million SNPs were incorporated in the GWAS meta-analysis, with genotyping specifics detailed in earlier studies^18,20^. Supplementary Table 1 compiles all 486 metabolites.

### Outcome sources of iridocyclitis

Summary statistics for iridocyclitis GWAS were obtained from the FinnGen research project (https://r9.finngen.fi/)^21^. Iridocyclitis was classified based on the International Classification of Disease, Ninth Revision (ICD-9; 364.00) and ICD-10 (H20.0) codes. However, the database does not segregate the iridocyclitis cases into distinct categories based on etiology. Consequently, our analysis included all cases of iridocyclitis as a single group, without distinguishing between infectious and non-infectious origins. In our analysis of iridocyclitis, which covered 7306 cases and 357,814 controls, key adjustments were made for variables including age, sex, genetic relatedness, and genotyping batch. Importantly, the study also incorporated adjustments for the first 10 principal components, adhering to FinnGen's GWAS data protocol^21^. This approach corrects for population structure peculiar to the Finnish cohort, ensuring our methods are in harmony with FinnGen standards and enhancing the comparability of our findings with related studies.

### Selection and validation of instrumental variables

Our study rigorously adhered to three foundational assumptions in the selection of IVs for MR: (1) pertinence to metabolite exposure, (2) association with the outcome solely through the exposure, and (3) absence of correlation with confounding factors. Genetic variations linked to the 486 metabolites were initially isolated, adhering to a significance threshold of *p* < 1 × 10^–5^. Further refinement of independent variants utilized a clumping methodology, establishing a linkage-disequilibrium threshold of r^2^ < 0.01 within a 500 kb spatial parameter. Ensuing elimination dismissed palindromic SNPs with middle allele frequency (MAF) and SNPs manifesting incongruent allelic effects (e.g., A/G versus A/C). To validate the efficacy of the selected IVs, we examined the variance explained (R^2^) and F statistic parameters, adhering to a minimal threshold of F > 10. Consideration of bias mitigation guided instrument selection, discarding variants demonstrating suboptimal statistical robustness and excluding outcome-related SNPs from the IVs (*p* < 1 × 10^–5^). To meet the third assumption, the IVs were meticulously pruned to include only those associated with metabolites, devoid of direct linkage with the outcome (*p* < 1 × 10^–5^). Ultimately, metabolites concomitant with more than four SNPs were nominated for MR examination. This comprehensive methodology underpinned the validation of the IVs, augmenting the dependability of our MR investigation.

### Mendelian randomization analysis

We applied an exhaustive two-sample MR methodology to metabolites symbolized by at least four independent IVs. This stratagem facilitated statistical examination and refinement for possible pleiotropy. The primary analysis employed the multiplicative random-effect inverse variance weighted (IVW) technique, conducting a meta-analysis that integrated Wald approximations from individual SNPs. This approach provided a comprehensive effect estimation of the influence of each metabolite on iridocyclitis^22^. To augment our trust in these determinations, we invoked two supplementary methodologies, specifically, the Weighted Median (WM) and MR-Egger, both of which exhibited resilience under lenient conditions. We selectively applied these to metabolites that manifested significant estimations via IVW (*p* < 0.05). The WM method is accommodating of up to 50% of SNPs as invalid^23^, whereas MR-Egger proficiently identifies horizontal pleiotropy and heterogeneity, even amidst the ubiquity of universal horizontal pleiotropy^24^.

### Sensitivity analysis

Sensitivity analysis served as an instrumental component in scrutinizing the detrimental implications of horizontal pleiotropy and heterogeneity on our MR estimations. To counteract these effects, we employed a multifaceted array of assessments: the Cochran's Q test for the unearthing of heterogeneity^25^, MR-Egger intercept examination for the discernment of directional pleiotropy and concomitant biases^26^, and MR-PRESSO^27^ coupled with Radial MR^28^ to isolate and rectify outliers and heterogeneous SNPs. Subsequently, we executed a leave-one-out evaluation to ascertain that no individual SNP exerted a disproportionate sway on our MR approximations^29^.

Thus, the causal impact of blood metabolites on iridocyclitis was authenticated through a meticulous procedure encompassing: (1) a consequential *p*-value in the seminal IVW-mediated analysis (*p* < 0.05), (2) a nonexistence of discernible heterogeneity or horizontal pleiotropy, and (3) an absence of substantive modifications in MR estimations attributable to a solitary SNP.

### Investigating genetic correlation and direction of causality

In a bid to address the potential genetic correlation between exposure and outcome variables, which may adulterate MR estimations, we instituted ancillary analyses^30,31^. Despite the exclusion of iridocyclitis related SNPs during the selection of instrumental variables, unrelated SNPs might fortuitously impinge upon iridocyclitis genetics. To counteract this potential complication, we harnessed LDSC regression. This method assesses the co-inheritance of two traits through SNP-based Chi-squared statistics, thereby ensuring that any perceived causal effects are unclouded by coheritability.

Moreover, to delve into the directionality of causality and gauge the impact of iridocyclitis phenotypes on putative metabolites, we orchestrated a reverse MR analysis. This stratagem contributes to the eradication of reverse causality bias, thereby fortifying our comprehension of the orientation and magnitude of causal relationships.

## Results

### Assessment of strength and validity in instrumental variables

In our investigation, we orchestrated a two-sample MR analysis to evaluate the causal effect of 486 serum metabolites on iridocyclitis, drawing upon GWAS summary data hailing from the FinnGen project. Of these metabolites, six (namely fructose, xanthione, glutamate, ergothioneine, X-11843, and X-12776) were characterized by fewer than four IVs and were consequently omitted from the analysis. Accordingly, we synthesized IVs for the residual 480 metabolites, with the count of SNPs varying from 4 to 477 for each metabolite (Supplementary Table 1). These IVs encapsulated a variance spanning from 0.002 to 0.708% within their corresponding metabolites. Additionally, the F statistics for all SNPs affiliated with metabolites exceeded 10, thereby signaling a robust potency of the IVs.

### Investigating the connection between metabolites and iridocyclitis

**Figure 2:**
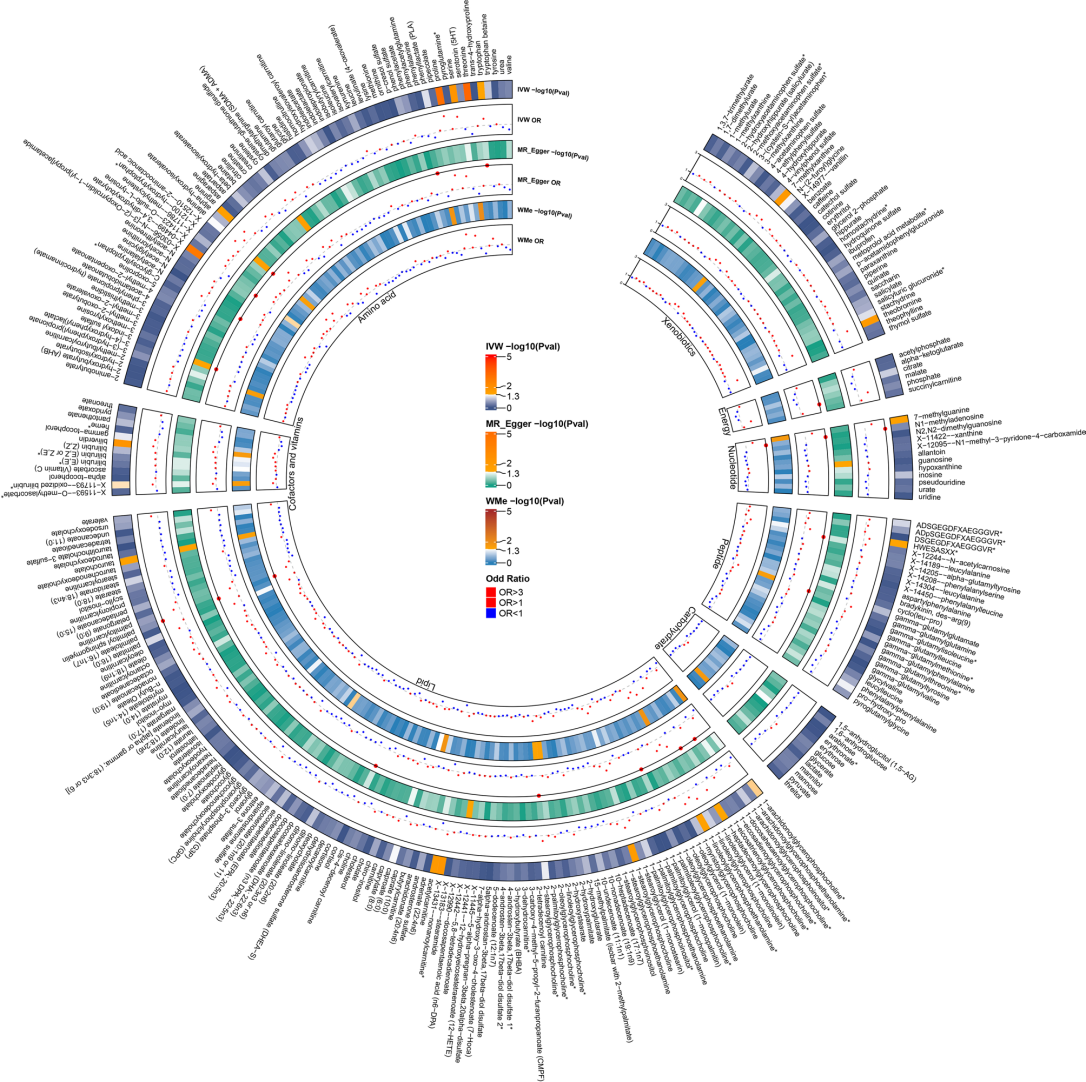
Circular heatmap of Mendelian randomization analysis results for serum metabolites in iridocyclitis. This figure illustrates the causal associations between 309 identified serum metabolites and iridocyclitis, as determined by MR analysis. Each concentric circle represents a different MR method: the outermost circle for inverse variance weighted (IVW), followed by MR-Egger, and the innermost for weighted median (WMe). The significance of each metabolite's association with iridocyclitis is shown by the negative logarithm of the p-value (−logP), with higher values indicating greater significance. Dots along the circles depict Odds Ratios (ORs): red for ORs > 1 (indicating potential risk factors), green for ORs < 1 (suggesting potential protective factors), with notably larger dots highlighting metabolites of significant interest. Distinct segments represent different categories of metabolites for easy identification. Metabolites with insufficient instrumental variables (fewer than four) are marked in grey and excluded from detailed analysis.

The IVW method was harnessed to explore causal relationships between 480 metabolites and iridocyclitis, utilizing GWAS summary data. We ascertained 23 robust causative associations (*p* < 0.05), comprising 17 identified metabolites and 6 of an as-yet undefined nature (Fig. 2, Supplementary Table 2). Focusing our subsequent analyses on these 17 acknowledged metabolites, we acknowledged the potential vulnerability of the IVW method to weak instrument bias despite its effectiveness in discerning causal links. To bolster our inferences, additional sensitivity and pleiotropy evaluations were performed. Through Cochran's Q test, we discerned notable heterogeneity in acetylcarnitine (*p* < 0.05). Furthermore, the employment of MR-Egger regression allowed for a careful examination of horizontal pleiotropy, revealing no detectable pleiotropic effects among the known metabolites.

Following the primary analysis, we leveraged the MR-PRESSO method to delve deeper into the heterogeneity, revealing the presence of outliers in acetylcarnitine (*p* < 0.05). When these outliers were removed, the MR-PRESSO findings corroborated the existence of heterogeneous SNPs (Supplementary Table 3), consistent with earlier insights gleaned from Cochran's Q test and MR-Egger regression. Additionally, bidirectional Mendelian randomization confirmed that reverse causality supported the causal relationship between most of the metabolites and iridocyclitis (*p* > 0.05), with a significant exception being 7-methylxanthine, which was excluded due to pronounced pleiotropy (Supplementary Tables 4 and 5). Furthermore, after meticulously excluding heterogeneity and pleiotropy, the reverse Mendelian randomization of HWESASXX (OR = 1.015, 95% CI = 1.002–1.028, *p* = 2.48 × 10^–2^) was identified as a risk factor. When considered in conjunction with the positive Mendelian findings, our results suggest a potential association between certain metabolites and iridocyclitis. However, it is important to emphasize that these findings do not definitively establish a direct impact on the progression of the disease. Further research, particularly longitudinal studies, is required to explore the nature of these associations and their implications for disease progression more thoroughly.

### Investigating the specific metabolite connections in iridocyclitis

**Figure 3:**
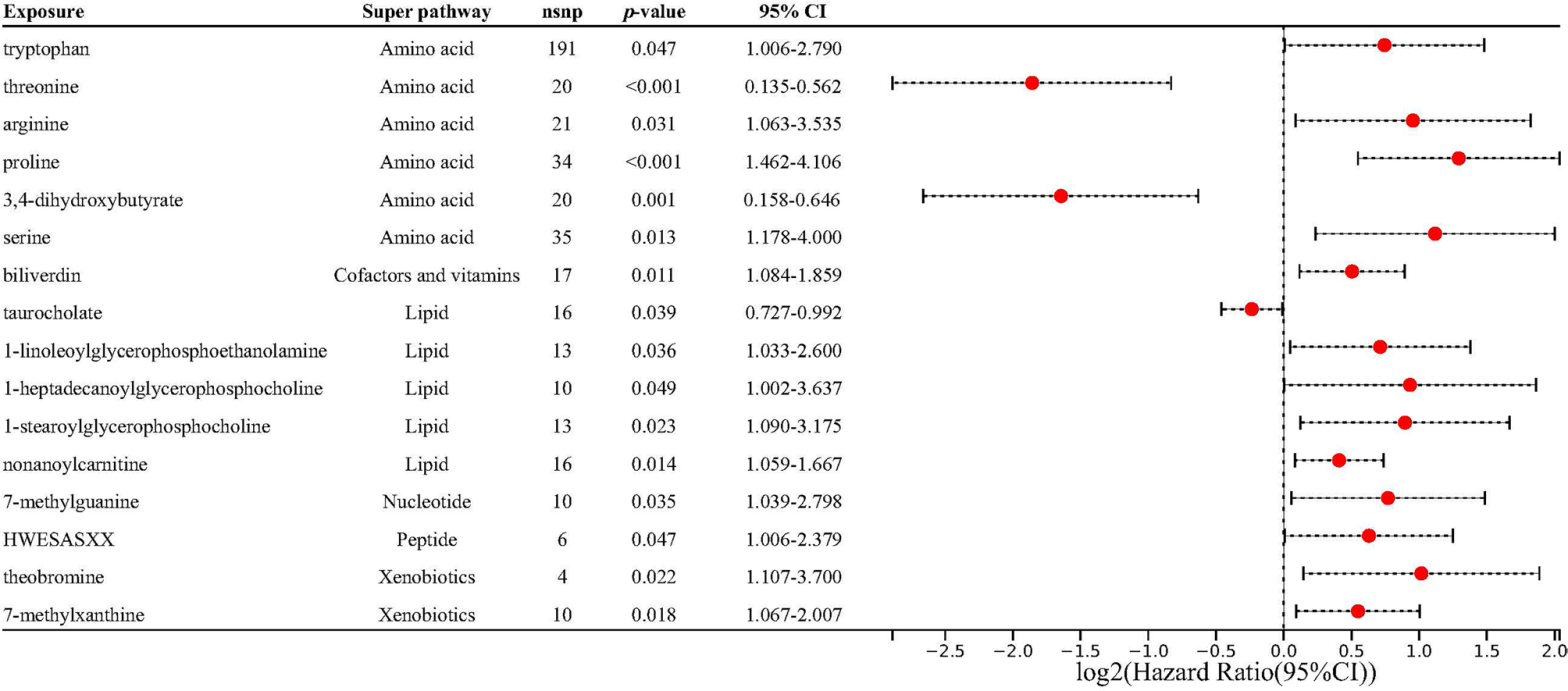
Forest plot showing causal estimates between serum metabolites and iridocyclitis. Odds ratio and 95% CI were obtained using the inverse variance weighting method. OR values less than 1 were protective of causal effects, and OR values greater than 1 were causative of pathogenic effects. *OR* odds ratio, *CI* confidence interval, *nsnp* number of single nucleotide polymorphisms.

Upon careful elimination of horizontal pleiotropy and heterogeneity's effects, we pinpointed unique associations between 16 particular metabolites and the risk of iridocyclitis through the IVW method (Fig. 3). This investigation into iridocyclitis encompassed an examination of various metabolites across diverse pathways to identify both risk and protective factors. In the amino acid pathway, risk factors identified include tryptophan (OR = 1.675, 95% CI = 1.006–2.790, *p* = 4.72 × 10^–2^), arginine (OR = 1.938, 95% CI = 1.063–3.535, *p* = 3.09 × 10^–2^), proline (OR = 2.450, 95% CI = 1.462–4.106, *p* = 6.69 × 10^–4^), serine (OR = 2.170, 95% CI = 1.178–4.000, *p* = 1.30 × 10^–2^), and protective factors include threonine (OR = 0.276, 95% CI = 0.135–0.562, *p* = 3.94 × 10^–4^) and 3,4-dihydroxybutyrate (OR = 0.320, 95% CI = 0.158–0.646, *p* = 1.48 × 10^–3^). The cofactors and vitamins pathway revealed biliverdin as a risk factor (OR = 1.419, 95% CI = 1.084–1.859, *p* = 1.09 × 10^–2^). In xenobiotics, risk factors include theobromine (OR = 2.024, 95% CI = 1.107–3.700, *p* = 2.20 × 10^–2^) and 7-methylxanthine (OR = 1.463, 95% CI = 1.067–2.007, *p* = 1.83 × 10^–2^). Within the lipid pathway, taurocholate was identified as a protective factor (OR = 0.849, 95% CI = 0.727–0.992, *p* = 3.89 × 10^–2^), whereas 1-linoleoylglycerophosphoethanolamine (OR = 1.639, 95% CI = 1.033–2.600, *p* = 3.61 × 10^–2^), 1-heptadecanoylglycerophosphocholine (OR = 1.909, 95% CI = 1.002–3.637, *p* = 4.92 × 10^–2^), and 1-stearoylglycerophosphocholine (OR = 1.860, 95% CI = 1.090–3.175, *p* = 2.29 × 10^–2^) were found as risk factors. Additionally, the peptide HWESASXX (OR = 1.547, 95% CI = 1.006–2.379, *p* = 4.70 × 10^–2^), nucleotide 7-methylguanine (OR = 1.705, 95% CI = 1.039–2.798, *p* = 3.48 × 10^–2^), and lipid nonanoylcarnitine (OR = 1.328, 95% CI = 1.059–1.667, *p* = 1.42 × 10^–2^) were also identified as risk factors for iridocyclitis. These findings provide significant insights into the associations between metabolites and iridocyclitis.

### Evaluating genetic influence on iridocyclitis and metabolites

In the LDSC analyses assessing the genetic underpinning of various metabolites, several showcased significant heritability. Most notably, tryptophan displayed an exceptionally high heritability with an h^2^ value of 0.975 (*p* = 1.06 × 10^–43^). Proline also manifested a notable genetic influence, as indicated by its h^2^ of 0.429 (*p* = 2.32 × 10^–10^). Other metabolites showing significant SNP heritability included 3,4-dihydroxybutyrate (h^2^ = 0.173, *p* = 1.61 × 10^–10^), theobromine (h^2^ = 0.158, *p* = 5.30 × 10^–3^), 1-heptadecanoylglycerophosphocholine (h^2^ = 0.154, *p* = 2.01 × 10^–2^), and 7-methylxanthine (h^2^ = 0.166, *p* = 4.79 × 10^–2^). Several metabolites, such as 1-linoleoylglycerophosphoethanolamine, HWESASXX, and 7-methylguanine could not have their heritability reliably estimated, as evidenced by NA values (Supplementary Table 6).

In the subsequent stage of our investigation, we probed the genetic correlation between iridocyclitis and six metabolites. Notably, the majority of these metabolites revealed no substantial genetic correlation with iridocyclitis (*p* > 0.05); the substances encompassed tryptophan, proline, 3,4-dihydroxybutyrate, theobromine, and 7-methylxanthine (Supplementary Table 7). Such insight intimates that a confounding effect emanating from shared genetic foundations on our MR assessments is unlikely. Of particular significance is the presence of 1-heptadecanoylglycerophosphocholine (*p* < 0.05), indicative of a genetic correlation with iridocyclitis. These observations lend additional substantiation to the integrity of our MR evaluations and underscore that the discerned correlations between these metabolites and iridocyclitis are less likely to be significantly swayed by mutual genetic underpinnings.

**Figure 4:**
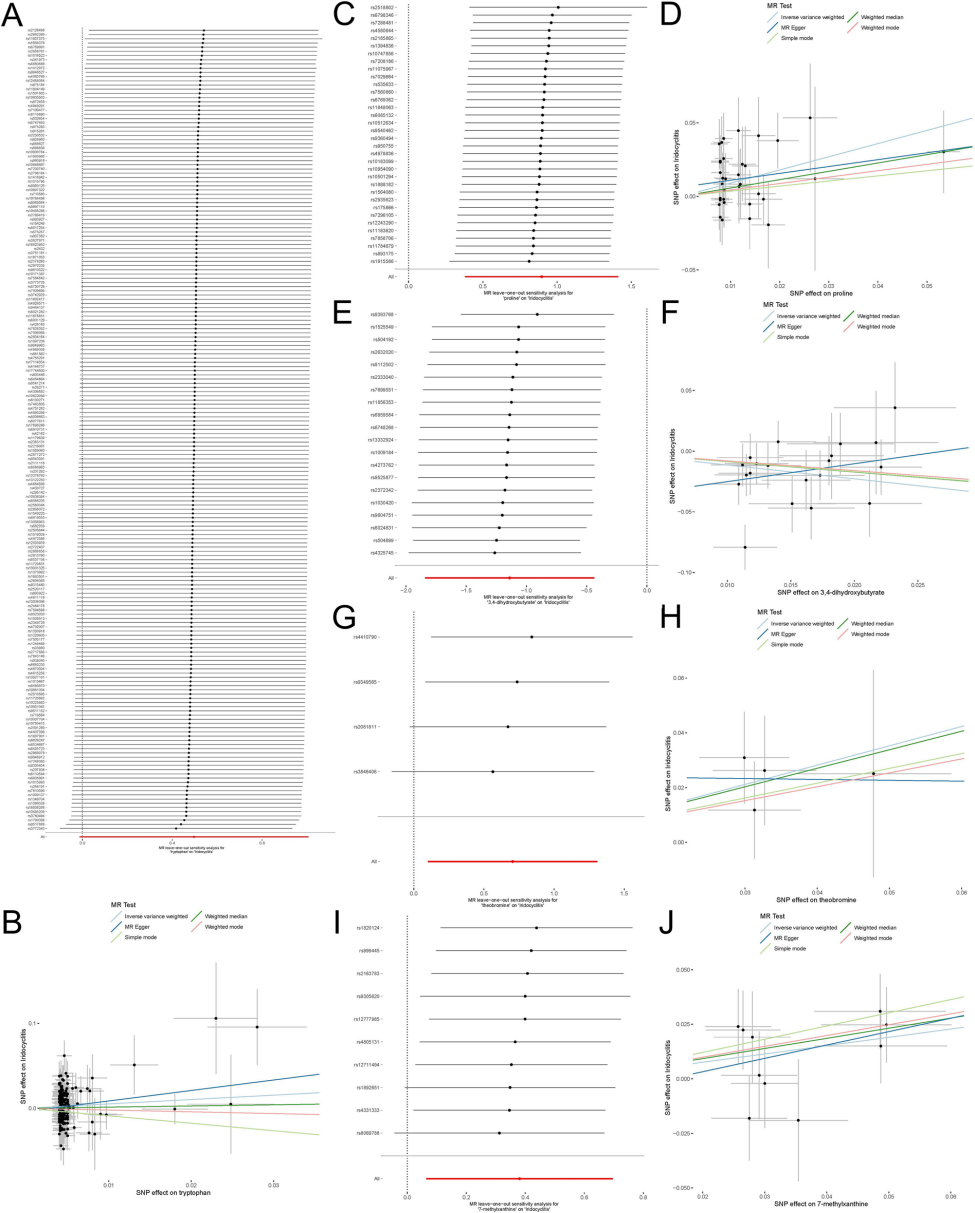
Causal association of five different serum metabolites with the risk of developing iridocyclitis. Leave-one-out analysis (**A**) and scatter plot (**B**) of the association between tryptophan and iridocyclitis. Leave-one-out analysis (**C**) and scatter plot (**D**) of the association between proline and iridocyclitis. Leave-one-out analysis (**E**) and scatter plot (**F**) of the association between 3,4-dihydroxybutyrate and iridocyclitis. Leave-one-out analysis (**G**) and scatter plot (**H**) of the association between theobromine and iridocyclitis. Leave-one-out analysis (**I**) and scatter plot (**J**) of the association between 7-methylxanthine and iridocyclitis. Leave-one-out analysis: used to assess whether any single instrumental variable drove the causal effect. Scatterplot: all 5 methods are described in this paper. Light blue, dark blue, light green, dark green, and pink lines represent inverse variance weighted (IVW), MR-Egger, simple mode, weighted median (WM), and weighted mode, respectively. *MR* Mendelian randomization, *SNP* single nucleotide polymorphism.

To further substantiate the causality between five metabolites and iridocyclitis, a methodical leave-one-out analysis was undertaken. The findings unequivocally revealed that the associations were not the result of an isolated SNP, but rather emanated from the collective influence of several SNPs (Fig. 4). Additionally, the conclusions drawn from each sensitivity examination were visually portrayed using scatter plots (Fig. 4), thereby furnishing an intuitive and pictorial comprehension of the analytical proceedings.

Within the context of our LDSC analysis, a considerable number of NAs in heritability and genetic correlation might emerge from constraints such as limited sample size, suboptimal SNP coverage, or intrinsic data quality issues. Despite these potential impediments, legitimate evaluations for specific attributes were discerned and meticulously discussed, shedding light on more profound biological nuances.

## Discussion

To our knowledge, this is the first study to explore the potential association between iridocyclitis and serum metabolites and its directionality. In this MR investigation, we investigated genetic relationships between serum metabolites and susceptibility to iridocyclitis using metabolomics data from two significant European cohorts. We created a precise technique to make the careful selection of IVs for MR analyses easier and to provide a reliable evaluation of how any one of 480 metabolites may affect iridocyclitis.

**Figure 5:**
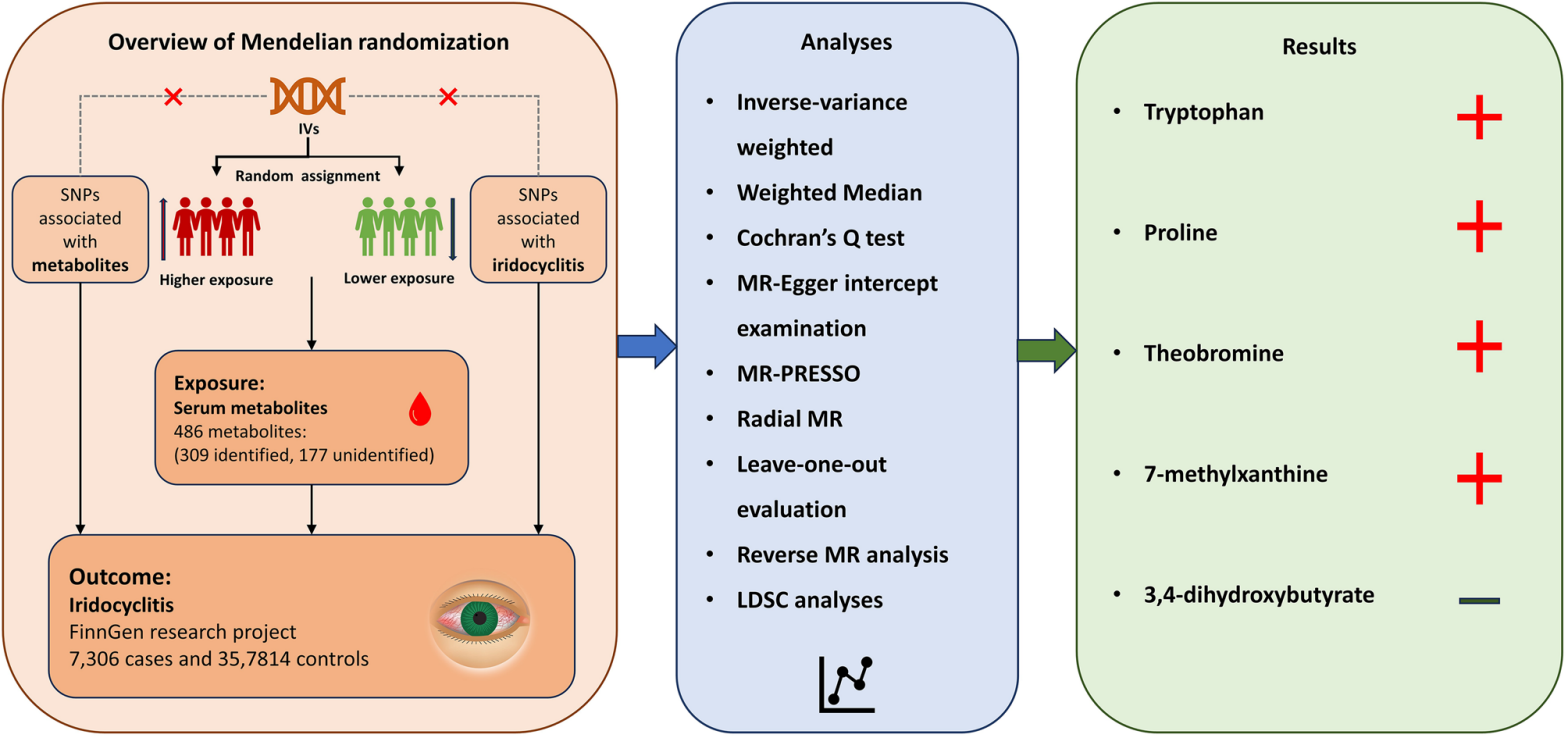
Schematic representation of the results of this Mendelian randomization study. A red plus sign indicates a risk factor and a green minus sign indicates a protective factor. *MR* Mendelian randomization, *LDSC* linkage disequilibrium score regression.

The present study examined the complex relationship between various metabolites and the risk of iridocyclitis, and also provided a preliminary exploration of the underlying pathologic mechanisms. Utilizing an IVW approach, we identified 23 metabolites associated with susceptibility to iridocyclitis. After a rigorous sensitivity analysis, 16 known metabolites remained significantly correlated. To insulate our findings from common genetic influences that might confound the true association, we used LDSC analysis, which detected six metabolites (tryptophan, proline, 3,4-dihydroxybutyrate, theobromin, 1- heptadecanoylglycerophosphocholine, and 7-methylxanthine) with a significant degree of heritability. Also, except for 1-heptadecanoylglycerophosphocholine, the other five metabolites did not share any common heritability with iridocyclitis (Fig. 5).

In addition to serving as essential protein building blocks, amino acids are necessary for the immune system to work properly. Tryptophan, arginine, proline, and serine have been identified as risk factors for iridocyclitis, whereas threonine has been suggested as a protective factor. These results suggest that imbalances in the metabolism of these amino acids may play a role in the immunopathogenesis of iridocyclitis. It is believed that a key process for determining immune cell responsiveness is arginine metabolism^32,33^. Changes in arginine's biosynthetic pathway may highlight T-cell dysfunction during the iridocyclitis disease process. Arginine can promote the survival of activated T cells and the production of central memory-like cells by inducing metabolic changes between glycolysis and oxidative phosphorylation^34,35^. Indoleamine 2,3-dioxygenase 1 (IDO-1) is thought to inhibit the proliferation of microbes, tumor cells, and activated T lymphocytes, possibly by catabolizing the necessary amino acid tryptophan. IDO-1 is expressed at low levels in healthy typical individuals, but expression is significantly increased in infection or inflammation caused by lipopolysaccharide, proinflammatory cytokines, or other factors^36,37^. Imbalance of the inflammatory response is closely related to the pathogenesis of iridocyclitis, and further ex vivo and in vivo studies are needed to explore the specific roles of these metabolites in iridocyclitis, which may provide new strategies for treatment. Lipid metabolism has an important role in immune regulation and may be associated with the development of certain autoimmune diseases^38,39^. In our study, taurocholate was a protective factor against iridocyclitis, whereas 1-linoleoylglycerophosphoethanolamine, 1-heptadecanoylglycerophosphocholin, and 1- stearoylglycerophosphocholine, on the other hand, lead to an increased risk of the disease. Previous studies have shown that serum oleic acid levels, urinary palmitic acid and oleic acid levels are significantly higher in patients with Behçet’s disease^40,41^, and in patients with VKH, sweat palmitic acid levels, serum oleic acid levels, and atrial chondroitidic acid levels are significantly higher than in controls^42,43^. The elevated levels of serum lipid metabolites in iridocyclitis suggest that they may play an important role in its pathophysiology, and further studies are needed to investigate the functions of these lipids and their roles in the pathogenesis of iridocyclitis, potentially leading to the discovery of new therapeutic targets.

In our Mendelian randomization study, we conducted a heritability analysis on metabolites associated with iridocyclitis, finding significant heritability for tryptophan, proline, 3,4-dihydroxybutyrate, theobromine, 1-heptadecanoylglycerophosphocholine, and 7-methylxanthine. This implies a genetic influence on their levels. Nonetheless, except for 1-heptadecanoylglycerophosphocholine, these metabolites did not exhibit a direct genetic correlation with iridocyclitis. Such results highlight that although these metabolites' levels are genetically influenced, they may not directly impact iridocyclitis through genetic mechanisms, suggesting their potential indirect role in the disease's progression rather than as direct genetic contributors. Furthermore, given the heritable nature of these metabolites, preventive trials exploring dietary or lifestyle changes in at-risk populations could offer insights into iridocyclitis prophylaxis. Such interventions, based on modifying levels of specific metabolites, would have the potential to prevent or ameliorate the development of iridocyclitis. While our MR approach has laid the groundwork for understanding the genetic relationships between serum metabolites and iridocyclitis, we emphasize that clinical trials are essential for translating these associations into practical treatment strategies. The interventional studies would not only confirm the causality inferred from our MR findings but could also uncover new therapeutic approaches for iridocyclitis management.

There are a few restrictions on this study. Firstly, we acknowledge a critical limitation related to our primary data source: the FinnGen research database does not provide detailed classifications for the various subtypes of uveitis, categorizing cases as a single entity without subtype distinctions. Despite this, we focused on iridocyclitis, the most common form of uveitis, due to its prevalence and significant impact on patient quality of life. In order to verify and prove causation, additional experiments and studies are required because the links found are based on statistical associations. Despite taking steps to locate and remove anomalous single nucleotide polymorphisms, we cannot entirely rule out the influence of potential heterogeneity on the results. This approach ensured a homogeneous study population and minimized the inherent heterogeneity of broader uveitis categories. Our Mendelian randomization study focuses on the genetic determinants of serum metabolites' impact on iridocyclitis risk. However, it's crucial to recognize that this genetic focus does not fully encompass the potential role of environmental factors. These factors, which may include lifestyle choices, infections, and exposure to pollutants, can also significantly influence iridocyclitis risk. Future research should aim to integrate both genetic and environmental data to provide a more complete understanding of the disease's etiology.

Our study elucidates the complex interactions between metabolites and iridocyclitis, revealing the protective role of threonine and taurocholate, as well as tryptophan, arginine, 1-linoleoylglycerophosphoethanolamine, 1-heptadecanoylglycerophosphocholin as risk factors for the disease. These results not only provide an aid in the diagnosis of the disease and help to investigate the pathophysiological mechanisms involved in the development of iridocyclitis, but may also suggest the prevention of iridocyclitis as well as personalized treatment of the disease.

### Supplementary Information

Supplementary Tables.

## Data Availability

The original contributions presented in the study are included in the article/supplementary material. Further inquiries can be directed to the corresponding author.
